# Insulin Resistance and High Blood Pressure: Mechanistic Insight on the Role of the Kidney

**DOI:** 10.3390/biomedicines10102374

**Published:** 2022-09-23

**Authors:** Gabriele Brosolo, Andrea Da Porto, Luca Bulfone, Antonio Vacca, Nicole Bertin, Laura Scandolin, Cristiana Catena, Leonardo A. Sechi

**Affiliations:** Clinica Medica, Department of Medicine, University of Udine, 33100 Udine, Italy

**Keywords:** animal models, hypertension, insulin receptors, kidney, messenger RNA, sodium

## Abstract

The metabolic effects of insulin predominate in skeletal muscle, fat, and liver where the hormone binds to its receptor, thereby priming a series of cell-specific and biochemically diverse intracellular mechanisms. In the presence of a good secretory reserve in the pancreatic islets, a decrease in insulin sensitivity in the metabolic target tissues leads to compensatory hyperinsulinemia. A large body of evidence obtained in clinical and experimental studies indicates that insulin resistance and the related hyperinsulinemia are causally involved in some forms of arterial hypertension. Much of this involvement can be ascribed to the impact of insulin on renal sodium transport, although additional mechanisms might be involved. Solid evidence indicates that insulin causes sodium and water retention, and both endogenous and exogenous hyperinsulinemia have been correlated to increased blood pressure. Although important information was gathered on the cellular mechanisms that are triggered by insulin in metabolic tissues and on their abnormalities, knowledge of the insulin-related mechanisms possibly involved in blood pressure regulation is limited. In this review, we summarize the current understanding of the cellular mechanisms that are involved in the pro-hypertensive actions of insulin, focusing on the contribution of insulin to the renal regulation of sodium balance and body fluids.

## 1. Introduction

It has been more than 30 years since seminal clinical investigations demonstrated that individuals with arterial hypertension have reduced sensitivity to insulin and hyperinsulinemia in comparison with subjects with normal blood pressure [1,2]. Clinical investigators noted also that most people with insulin resistance and a high plasma insulin concentration had hypertension as a part of a cluster of metabolic conditions that included being overweight, increased fasting glucose, and dyslipidemia [2,3]. These observations led to the development of the concept of “metabolic syndrome” as a condition that is closely associated with an increased risk of cardiovascular events and recognizes insulin resistance as a key underlying factor [4,5]. Seminal support for the concept of a functional link between insulin and blood pressure came from studies that reported that insulin resistance was correlated with blood pressure in nonobese hypertensive subjects [1] and that hyperinsulinemia was a distinctive feature of subjects with essential, but not secondary hypertension [6]. Moreover, the possibility that insulin resistance and hyperinsulinemia are not the consequence of high blood pressure, but instead result from a preexisting condition, was suggested by the observation of abnormal glucose metabolism in the normotensive offspring of hypertensive patients [7].

The causes of essential hypertension are not known, but excess body fat appears to be the main culprit. Results of studies conducted in different populations indicate that approximately 70% of the risk for essential hypertension can be ascribed to being overweight [8]. Additionally, the body distribution of fat seems to be relevant to the impact of obesity on blood pressure and related metabolic conditions, since robust data indicate that visceral fat is much more closely associated with the risk of metabolic conditions and hypertension than subcutaneous fat [9]. The factors responsible for blood pressure increase in obese patients are still under debate, and many pathophysiological mechanisms have been potentially implicated [10], including proinflammatory changes, oxidative stress, lipotoxicity, and mitochondrial dysfunction [11]. In this context, an overriding role has been attributed to insulin resistance and hyperinsulinemia and the renal effects of increased circulating insulin levels. Evidence obtained both in the experimental and clinical setting clearly indicates that insulin promotes renal sodium retention leading to volume expansion, an effect that is preserved in states of metabolic insulin resistance. In this narrative review, after a brief summary of the current knowledge of the general mechanisms that modulate insulin sensitivity, we examine the contribution of insulin resistance and hyperinsulinemia to hypertension in humans and experimental animals, by specifically focusing on the renal actions of insulin and its role in the regulation of sodium balance and body fluids.

## 2. Insulin Resistance: General Concepts

Insulin resistance identifies any condition in which the response of metabolic target tissues, namely, skeletal muscle, fat, and liver, to normal circulating levels of insulin is impaired [12]. Insulin resistance is relatively common, mostly occurring in patients with type 2 diabetes, obesity, metabolic syndrome, polycystic ovary syndrome, and arterial hypertension. Because of the close association with hypertension and atherosclerosis-related cardiovascular events, insulin resistance has triggered extensive research both in the medical and biological fields [13,14]. Nonetheless, the cellular mechanisms linking arterial hypertension and atherosclerosis to insulin resistance are still under investigation [15,16].

The expression “insulin resistance” as commonly considered in the clinical setting refers to the inability of insulin to maintain normal glucose homeostasis. This invariably leads to increased production of insulin (compensatory hyperinsulinemia) in the attempt to facilitate glucose uptake by target tissues, thereby preserving normal blood glucose levels [17]. Therefore, the presence of insulin resistance implies the existence of an inappropriate effect of the hormone on glucose homeostasis and other metabolic targets such as inhibition of lipolysis but neglects many other facets of insulin action. For these reasons, it becomes important to separate the effects of insulin resistance from those of hyperinsulinemia because excess circulating insulin might cause overstimulation of tissues that are marginally involved in metabolism and have preserved sensitivity to insulin action.

Cloning of the insulin receptor and unraveling of the intracellular signaling systems primed by insulin opened a new era in the understanding of mechanisms of insulin resistance [18,19]. The insulin receptor is encoded by a single gene of 22 exons located on human chromosome 19 that generates a single chain pro-receptor that, in turn, undergoes glycosylation, dimerization by disulphide bonds, and cleavage to two different subunits. As a result of processing, the insulin receptor is a disulphide-bond-linked tetramer composed of two alpha and two beta subunits. Recently, visualization of the three-dimensional structure of the insulin receptor has been made possible by the use of X-ray crystallography associated with electron microscopy [20]. The alpha subunit resides entirely on the outer surface of the cell and contains the insulin binding site. Two insulin binding sites, one with high affinity and one with low affinity, are in the alpha subunit [21]. Alternative splicing of the Exon 11 of the insulin receptor gene leads to the existence of two different receptor isoforms with different affinity to insulin and other homologous ligands such as insulin growth factor-1 and -2 [22]. The beta subunit is a transmembrane protein and contains a domain that spans the cell membrane and transduces the signal to the interior of the cell by virtue of its tyrosine kinase activity [23]. After insulin binding, tyrosine residues of the intracellular domain of the beta subunit are phosphorylated (autophosphorylation) and the kinase intrinsic to this region is activated. After autophosphorylation, the kinase is further activated toward other intracellular substrates.

Two main insulin-related intracellular signaling pathways have been identified [18,19,20]. The first pathway involves the phosphorylation of insulin receptor substrates (IRS) [24], a family of homologous proteins that can be broadly expressed in tissues (IRS-1 and IRS2) or can be tissue- and/or species-restricted (IRS-3 and IRS-4) [25]. Phosphorylation of IRS triggers activation of phosphatidylinositol 3-kinase (PI3K) that initiates a chain of events that are directly involved in the metabolic and mitogenic effects of insulin. The most important among these events is the generation of phosphatidylinositol trisphosphate (PIP3) that is required to activate the serine/threonine kinase AKT, the final effector of most metabolic actions of insulin. The second pathway involves the activation of many intracellular substrates among which mitogen-activated protein kinases (MAPK: Erk 1 and 2) have a predominant role being involved in the control of mitogenic effects of insulin, but minor, if not any, relevance for the metabolic action. Thus, while impairment of the insulin signal along the IRS/PI3K pathway can explain the untoward metabolic impact of insulin resistance, the preserved response of the MAPK pathway in the presence of hyperinsulinemia might result in preserved or even increased ancillary, nonmetabolic effects of insulin [26]. 

Insulin sensitivity is under the influence of several factors such as age, ethnicity, body weight and fat content, dietary habits, physical exercise, and drugs [13], and its impairment is a common finding in many types of metabolic conditions, including obesity, type 2 diabetes, dyslipidemia, metabolic syndrome, and non-alcoholic fatty liver disease. Exogenous and endogenous cell factors have been listed as the possible underlying trigger elements of insulin resistance in these conditions. These factors include inflammatory mediators, adipocytokines, free radicals, and the microbiota on one hand, and mitochondrial dysfunction and endoplasmic reticulum stress on the other hand [15]. Over the past two decades, important insight has been provided as to changes in insulin receptors in terms of gene expression, number, and affinity to insulin, and abnormal intracellular signaling mechanisms could lead to the spectrum of phenotypes that are associated with insulin resistance [27]. Multiple abnormalities in the downstream signal transduction were reported including mutations of the IRS, defects in serine phosphorylation of IRS-1, activation of tyrosine phosphatases, and mutations of glucose transporters [15]. Specific tissue-directed knockouts or reconstitution of the insulin receptor in mice enabled investigators to expand the knowledge of the insulin resistance-associated conditions, by discriminating the relative contributions of different tissues to the insulin resistant state. Additionally, investigations of the complex network of intracellular signaling have led us to understand how insulin sensitivity can be impaired and the specific pathways that could be involved [28]. Although much is known about the mechanisms of insulin resistance in obesity and type 2 diabetes, the molecular links between insulin resistance and the multifaceted components of the metabolic syndrome including high blood pressure are only partially understood.

## 3. Insulin and the Kidney

It is now more than three decades that insulin resistance and the related hyperinsulinemia have been implicated as important contributors to blood pressure elevation in hypertensive patients [29]. Because studies conducted in experimental animals and humans provided evidence that insulin promotes renal sodium retention, important research efforts were directed to the study of this mechanism that, among the other pathways that could reasonably link insulin resistance to hypertension, appears to be overwhelmingly relevant.

### 3.1. Renal Insulin Receptors

**Figure 1:**
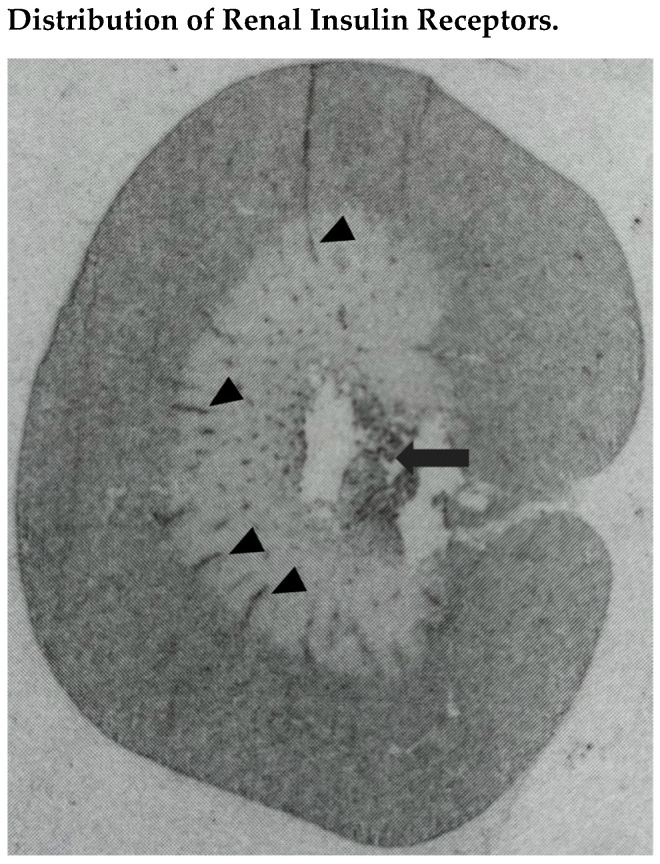
In situ-autoradiography showing distribution of radiolabeled insulin binding sites in the kidney of a normal Wistar-Kyoto rat. Insulin binding was greater in the renal cortex, where it was comparable in tubules and glomeruli that could not be distinguished from the surrounding tissue. In the renal medulla, radioligand binding was detected primarily in longitudinal structures crossing the outer portion, presumably corresponding to interlobar vessels (black arrowheads), and the inner portion, presumably corresponding to hilar fat (black arrow).

The presence of insulin receptors in the kidney was demonstrated with the use of different methodologies. The binding of ^125^I-labeled insulin to microdissected rat renal tubules was examined by Butlen et al. [30], reporting the greatest binding in the proximal convoluted and distal convoluted tubule, followed by the thick ascending limb and outer medullary collecting duct. We developed an in situ autoradiographic technique (Figure 1) to localize insulin receptors in the rat kidney, showing that radiolabeled insulin binding was greater in the renal cortex, where it was comparable in tubules and glomeruli that could not be distinguished from the surrounding tissue [31]. In the renal medulla, radioligand binding was detected primarily in longitudinal structures crossing the outer portion, presumably corresponding to interlobar vessels, and the inner portion, presumably corresponding to hilar fat. The same distribution of renal insulin receptors was reported in the chicken kidney which was shown to be upregulated by fasting [32]. In these experiments, the precise nature of the receptors identified by radiolabeled insulin was not entirely certain due to the possibility of nonspecific binding to other receptors that can be detected in the kidney, such as those for the insulin growth factor. Nonetheless, the demonstration of gene expression of the classic insulin receptor in whole homogenates of the rat kidney by detection of the specific messenger RNA (mRNA) further supported the evidence of the receptor presence [33]. Moreover, differential regulation of insulin receptor gene expression in tissues obtained from rats with experimentally-induced type 1 diabetes suggested a possible functional role for these receptors even in tissues uninvolved in metabolic processes [33]. Conclusive demonstration of the presence of insulin receptors in the rat kidney was obtained using immunoblotting and immunohistochemistry with specific antibodies directed to the alpha and beta subunits, showing expression of the receptor in the proximal tubule, thick ascending limb, distal convoluted tubule, and collecting duct [34].

### 3.2. Renal Effects of Insulin

The widespread distribution of insulin receptors along the renal tubule set the stage for a direct intervention of the hormone in the regulation of tubular sodium transport. In vitro studies demonstrated that insulin activates transmembrane sodium carriers [35], an effect that holds great relevance for nearly all tubular cells that are involved in salt reabsorption. Microperfusion studies of microdissected proximal tubules in rats demonstrated that insulin stimulates sodium reabsorption in this segment [36] by increasing the activity of the Na/H exchanger type 3 (NHE3) on the luminal side [37] and the Na-K ATPase on the basolateral side [38]. If not compensated by downstream transport, activated NHE3-mediated reabsorption significantly affects the overall renal sodium balance. Increased sodium transport in response to insulin was reported also in the isolated thick ascending limb of the Henle’s loop where insulin activates the furosemide-responsive Na-K-2Cl cotransporter (NKCC2) [39], an effect that was confirmed in humans with the use of the hyperinsulinemic-euglycemic clamp [40]. Last, insulin was demonstrated to activate the epithelial sodium channel (ENaC) [41] and Na-K ATPase [42] in cells of the distal tubule and collecting duct, and DeFronzo et al. [43] suggested that this effect on the last tract of the renal tubule is mostly relevant for the sodium retentive effects of insulin. In this study, the effects of exogenous insulin were examined in healthy subjects with the use of the hyperinsulinemic-euglycemic clamp, reporting a decrease of urine sodium excretion of approximately 50% in the absence of changes in renal plasma flow, glomerular filtration, or urine flow [43]. Similar results were obtained in two additional clamp studies conducted on healthy individuals. Skott et al. [44] reported a dose-dependent response of increasing plasma insulin concentrations in reducing sodium clearance. In this study, insulin infusion did not affect sodium reabsorption in the proximal tubule, while the distal fractional sodium reabsorption increased significantly. The same evidence of increased tubular sodium reabsorption at a distal nephron site was reported by Stenvinkel et al. [45] with the use of two different levels of hyperinsulinemia. Thus, experimental evidence indicates that insulin could activate mechanisms of renal sodium reabsorption across the entire renal tubule, while human hyperinsulinemic-euglycemic clamp studies suggest a preferential effect at a distal tubular site.

Additional strong evidence of the direct involvement of insulin in the modulation of renal sodium balance was obtained in experiments conducted in normal Sprague Dawley rats with manipulation of dietary sodium intake [46,47]. These rats were maintained with three different NaCl dietary contents for two weeks, and insulin receptor density and distribution were assessed by autoradiography. In the high-salt-fed rats, the binding capacity of the high-affinity insulin receptor was significantly reduced as compared to low-salt and normal-salt-fed rats, and regional analysis of insulin binding indicated that the high-salt diet reduced equally insulin binding across all tubular sites [46]. Additionally, quantification of insulin receptor mRNA showed an inverse relationship with salt intake in the absence of changes in plasma glucose and insulin levels [47]. In a series of elegant experiments, Ecelbarger and colleagues examined the relevance of insulin-mediated mechanisms for the regulation of renal tubular sodium transport by using renal tissue-directed knock-out. First, they generated mice with renal epithelial cell-specific knock-out of the insulin receptor expressing significantly lower levels of mRNA and protein in the renal cortex and medulla [48]. These knock-out mice had impaired natriuresis and higher blood pressure than wild-type animals when challenged with an oral sodium load. In later experiments, knock-out of the insulin receptor was specifically directed to the principal cells of the collecting duct, where sodium reabsorption is mediated by the ENaC [49]. While ENaC expression was comparable in knock-out and wild-type littermates, insulin exposure of split-open tubules turned on the ENaC activity in the wild-type but not the knock-out mice, suggesting that insulin regulates the activity of this sodium channel through binding to its own receptor. Moreover, indirect evidence of the role of the insulin receptor was obtained by blocking the activity of the ENaC with inhibitors of PI3K [49]. Finally, mice with selective insulin receptor knock-out in the collecting duct had blood pressure significantly lower than wild-type littermates and had blunted natriuretic response to benzamil, an ENaC antagonist [50]. When challenged with a high-salt diet the knock-out mice also showed reduced natriuretic response as compared to wild-type controls. Overall, the results of all these studies support the view of a fundamental contribution of insulin to the modulation of sodium and water balance and blood pressure through its renal receptor.

### 3.3. Renal Effects of Insulin in Diabetes

Many studies conducted in experimental animal models have demonstrated the functional relevance of renal insulin receptors in different pathological conditions associated with insulin resistance. Changes in the activity of renal sodium carriers were reported in rat models of type 1 diabetes [29] that are known to be associated with increased natriuresis/diuresis and extensive rearrangement of peptide receptor families in the kidney [51]. In the kidney of rats with alloxan-induced diabetes, Na-K-ATPase activity is significantly increased [52], and in streptozotocin-induced diabetes, the expression of the luminal sodium transport proteins from the thick ascending limb to the collecting duct of the nephron is greater than in controls [53,54]. In type 1 diabetes, these changes in tubular sodium transporters might have a compensatory function in reducing volume and sodium dispersion due to persistent glycosuria.

Changes in renal tubular sodium transport have been reported also in experimental animals with type 2 diabetes. In adult, insulin resistant, obese Zucker rats, Bickel et al. [55] reported a significantly lower activity of luminal sodium transporters in the proximal tubule, thick ascending limb, and collecting duct with decreased expression of NHE3, NKCC2, and ENaC. In addition, and most importantly, the administration of an insulin sensitizer (rosiglitazone) to obese Zucker rats improved glycemic control and normalized the expression of NKCC2 and ENaC [56]. Tiwari et al. examined by immunoblotting of whole kidney homogenates and immunocytochemistry the regulation of renal insulin receptor expression in diabetic rats with insulin resistance associated with either hyperinsulinemia (Zucker rats) or hypoinsulinemia (streptozotocin rats) [34]. The expression of insulin receptors was significantly reduced both in obese Zucker and streptozotocin-diabetic rats. In Zucker rats, renal insulin receptor levels were restored by administration of rosiglitazone and an inhibitor of the angiotensin II type 1 receptor (candesartan). Conversely, in streptozotocin-diabetic rats, correction of hypoinsulinemia with insulin treatment did not affect renal insulin receptor numbers. We examined insulin receptor regional binding and gene expression in the kidney of normal and streptozotocin-diabetic rats who were chronically infused with angiotensin II or were treated with an angiotensin-converting enzyme inhibitor (captopril) [57]. Insulin receptor density and mRNA levels were significantly greater in the kidney of diabetic rats, but neither angiotensin II infusion nor captopril administration affected either insulin receptor binding or gene expression.

In the last decade, newer drugs have been made available for the treatment of type 2 diabetes. Among these drugs, sodium-glucose transporter 2 (SGLT2) inhibitors have gained the stage because of their renoprotective effects [58], which are related to lowering of the intraglomerular pressure by modulation of pre- and post-glomerular vascular tone [59]. SGLT2 inhibitors lower blood glucose by increasing urinary glucose excretion with a mechanism that is apparently independent of insulin action [60]. While some studies have reported that surrogate indexes of insulin resistance such as the homeostasis model assessment (HOMA) and the Matsuda Index were improved by treatment of type 2 diabetes with SGLT2 inhibitors [60], studies conducted with the use of the hyperinsulinemic-euglycemic clamp provided controversial results [61,62]. To date, only a few studies have specifically investigated the effects of SGLT2 inhibitors on renal responsiveness to insulin. Ferrannini et al., through the use of the hyperinsulinemic clamp in healthy subjects, reported that the renal effects of insulin on sodium excretion are not abolished by SGLT2 inhibitors [63]. Conversely, Nizar et al. used a renal tubule-specific insulin receptor knock-out mice model to demonstrate that renal insulin receptor deletion significantly decreased SGLT2 expression, thereby increasing urinary glucose and sodium excretion and urine flow [64]. More studies are needed to better understand the interaction of SGLT2 and its inhibitors with the mechanisms of insulin-related antinatriuresis.

Thus, there is substantial evidence that insulin increases renal sodium reabsorption by acting on its receptors that are present throughout the renal tubule, leading to sodium retention and volume expansion. These tubular effects of insulin appear to be most relevant in the collecting duct, where the activity of the ENaC is directly linked to the activation of insulin receptors. The intervention of insulin in the regulation of renal sodium balance is altered in experimental models of diabetes. In this context, opposite changes in apical sodium transporters are detected in rats with hypoinsulinemic or hyperinsulinemic insulin-resistant diabetes, supporting the view that insulin contributes to these changes. Additionally, some data suggest the possibility that the activity of the renin-angiotensin system might contribute to the modulation of the renal tubular response to insulin.

## 4. Insulin Resistance in Hypertension

### 4.1. Association of Insulin Resistance with Essential Hypertension

The first study to suggest a pathophysiologic link between insulin and hypertension goes back to 1966 when Welborn et al. [65] reported higher plasma insulin concentrations in 19 essential hypertensive patients compared to normotensive controls. Approximately 20 years lapsed before this topic ended up again in the spotlight and many research groups could provide evidence that resistance to insulin-mediated glucose disposal is highly prevalent in patients with essential hypertension [1,2,6,66]. On the other hand, insulin resistance was broadly recognized as the underlying disorder of the “metabolic syndrome”, a condition that links together the pathophysiology of obesity, diabetes, dyslipidemia, and hypertension [67].

Although the hypothesis of the contribution of insulin resistance and the related hyperinsulinemia to hypertension has received robust support from several clinical and animal investigations, some population studies questioned this relationship [68]. In a multicentric study conducted on normotensive subjects, the European Group for the Study of Insulin Resistance (EGSIR) measured fasting plasma insulin, insulin-mediated glucose disposal, and blood pressure, reporting that blood pressure was correlated to both circulating insulin and insulin resistance, independently of demographic factors and degree of overweight [69]. Despite providing conclusive evidence of a significant link between insulin resistance, hyperinsulinemia, and blood pressure, the EGSIR study could not establish the causal relevance of the relationship. Therefore, it could be argued that hypertension leads to insulin resistance rather than the opposite, but this possibility was ruled out by many additional observations. First, secondary forms of hypertension are not associated with evidence of insulin resistance [70,71]. Second, hyperinsulinemia and insulin resistance have been demonstrated in normotensive siblings and offspring of hypertensive patients [72,73]. Third, and most importantly, prospective studies reported that insulin resistance/hyperinsulinemia anticipates the development of hypertension in normotensive individuals [74,75,76]. In a 10-year follow-up, Skarfors et al. [74] analyzed data of 2130 normotensive men, showing that baseline blood pressure, body mass index, and fasting and post-glucose load plasma insulin were independent predictors of progression to stable hypertension. Risk factors for the development of hypertension were also assessed in 278 normotensive women over a 12-year follow-up, reporting that fasting plasma insulin concentrations predicted transition from normal to high blood pressure independent of baseline body mass and weight changes [75]. Last, changes in blood pressure were investigated in Finnish children and adolescents over a 16-year period [76], and even in this study fasting insulin levels were directly related to blood pressure and predicted future blood pressure increase independently of body weight.

Despite the heavy evidence provided by the above studies as to the contribution of insulin to blood pressure increase, statistical interpretation of some population-based studies still raises residual skepticism around the existence of this relationship [77]. The main contention is related to the observation that, according to current criteria, less than 50% of hypertensive patients have impaired insulin sensitivity [78] and this might explain why in factor analysis blood pressure is disaggregated from the group of factors that are linked to insulin resistance [77]. In addition, independence from body fat mass of the relationship between insulin resistance/hyperinsulinemia and hypertension was questioned, because the possible contribution of visceral fat was not considered in most of the above studies [29]. This is important because previous evidence indicated that excess visceral fat conveys a higher risk for metabolic disorders and cardiovascular diseases than subcutaneous fat and greatly contributes to the metabolic syndrome [9]. Moreover, visceral fat could confound the correlation between insulin levels and blood pressure [79]. Visceral obesity might contribute to increased renal sodium reabsorption causing volume expansion by many possible mechanisms. These include activation of the renin-angiotensin-aldosterone system, increased activity of the sympathetic nervous system, physical compression of the kidney, and changes in circulating adipocytokines [8,29]. However, no direct assessment of the possible effects of visceral fat on renal responsiveness to insulin is currently available.

Nonetheless, the following three statements cannot be questioned and strongly support the contention of a substantial functional connection between insulin resistance and essential hypertension: (1) patients with essential hypertension have increased frequency of insulin resistance and hyperinsulinemia; (2) insulin resistance and hyperinsulinemia are detected in normotensive, first-degree relatives of essential hypertensive patients, but not in patients with secondary hypertension; and (3) prospective studies of normotensive individuals demonstrate that insulin resistance and hyperinsulinemia predict subsequent development of hypertension.

### 4.2. Mechanisms Linking Insulin Resistance with Hypertension: The Role of Sodium

Over the years, many experimental animal and clinical studies were designed to address mechanisms that possibly link insulin resistance and hyperinsulinemia to hypertension. As broadly defined, insulin resistance refers to a reduced insulin-mediated glucose uptake eliciting compensatory hyperinsulinemia that is needed to maintain glucose levels within normal limits. Elevated circulating insulin, in turn, might act on tissues that do not have direct metabolic involvement and have preserved insulin responsiveness, thereby triggering specific organ responses with potentially harmful effects. Some of these effects could hypothetically contribute to the blood pressure raise, including increased renal sodium reabsorption with volume expansion [80], activation of the sympathetic nervous system discharge with increased circulating catecholamines [81], and growth-promoting activity on vascular smooth muscle cells that, in association with endothelial dysfunction, might lead to increased vascular reactivity [10].

Many epidemiological investigations have implicated sodium homeostasis in the pathogenesis of hypertension [82]. Demonstration that some hypertensive patients have a significant raise in blood pressure when they eat a diet containing high sodium led to the development of the concept of salt-sensitive hypertension [83]. Following the demonstration that elevated circulating insulin causes renal sodium retention, it was suggested that hyperinsulinemia resulting from insulin resistance might have a role in the mechanisms of salt sensitivity in hypertension [84]. Studies conducted in hypertensive patients have shown an association of salt sensitivity with hyperinsulinemia and insulin resistance that is independent of age, gender, and body mass [84]. Therefore, among the possible pro-hypertensive mechanisms possibly linked to hyperinsulinemia, those related to the renal antinatriuretic effect of insulin appear to be those most relevant, and it was speculated that the result of insulin resistance and hyperinsulinemia might be the development of salt-sensitive hypertension [80].

According to this view, there is strong evidence that in the presence of resistance to the metabolic effects of insulin the renal sodium-retaining effect is not affected. This was initially suggested by classical studies that reported how a reduction of daily insulin doses in type 2 diabetic patients taking doses over their need because of a Somogyi phenomenon was associated with a significant increase in urinary sodium and water excretion and a decrease in body weight and blood pressure [85]. Preservation of the antinatriuretic effect of insulin in subjects with insulin resistance was also reported by Rocchini et al. [86], who examined the effects of insulin on renal sodium handling in obese and non-obese young adults with the hyperinsulinemic-euglycemic clamp. Due to insulin resistance, the amount of glucose needed to maintain euglycemia in obese subjects was four times lower than in non-obese subjects, but hyperinsulinemia decreased urinary sodium excretion comparably in the two groups. Similar findings were reported by Natali et al. [87] who compared the response to an oral glucose test in insulin-resistant hypertensive patients and normotensive controls. Post-glucose load glycemic profiles were comparable in the two groups, but plasma insulin response in hypertensive patients was two-fold that of controls, suggesting insulin resistance. However, following glucose-induced hyperinsulinemia, the absolute and fractional urinary excretion of sodium were comparably decreased in both groups. The same group subsequently compared hypertensive patients with normotensive controls with the use of the hyperinsulinemic-euglycemic clamp [88] and again, insulin sensitivity was found to be lower in hypertensive patients than in normotensive controls, while the decrease in urinary sodium excretion was comparable in the two groups. In addition, a comparison of obese and non-obese hypertensive patients included in this study who had significantly different tissue sensitivity to insulin showed an equal effect of hyperinsulinemia on sodium handling. The effects of hyperinsulinemia on renal sodium handling were further compared by using the hyperinsulinemic-euglycemic clamp on type 2 diabetic patients with comparable metabolic control but different degrees of insulin resistance and matched non-diabetic controls [89]. As expected, there was a significant difference in insulin sensitivity between the three groups, but the urinary sodium excretion rate decreased to a comparable extent in all three groups, further supporting the conclusion that the antinatriuretic effect of insulin is independent of the metabolic consequences of insulin resistance.

The evidence on the association between insulin resistance/hyperinsulinemia and renal sodium handling obtained in human studies was further expanded by the findings of experimental animal studies. Insulin receptor binding and insulin receptor gene expression were examined in Sprague Dawley rats that were fed a low-, normal-, or high-sodium diet for 14 days [46,47]. Analysis of insulin receptor binding showed that the high-sodium diet was associated with decreased receptor number in all renal sites. Assessment of insulin receptor gene expression by the measurement of mRNA levels consistently demonstrated a dose-dependent, inverse relationship with dietary sodium content, showing that the sodium-related renal insulin receptor down-regulation is mediated by decreased gene expression. Modulation of renal insulin receptor density is fundamental to determining the renal responsiveness to circulating insulin. Therefore, these findings indicate the existence of a feedback mechanism according to which the antinatriuretic effect of insulin is reduced when dietary sodium content is increased, and extracellular fluid volume is expanded. 

### 4.3. Molecular Mechanisms of Insulin Resistance in Hypertension

As stated above, much is known about the molecular mechanisms of insulin resistance in the principal clinical conditions that are associated with decreased insulin sensitivity. These mechanisms in hypertension are debated and current knowledge comes from studies conducted in experimental animal models in which hypertension was shown to be associated with insulin resistance [90]. Among these animal models, both the spontaneously hypertensive (SHR) and Dahl hypertensive (Dahl) rat have genetically-determined hypertension that requires high dietary salt to develop, whereas another, the fructose-fed rat, is induced by chronic fructose feeding [91].

#### 4.3.1. Spontaneously Hypertensive Rats

Abnormalities of glucose metabolism, including insulin resistance and hyperinsulinemia have been reported in SHR in comparison to their Wistar-Kyoto controls (WKY) [92,93]. Moreover, blood pressure is under the influence of dietary sodium intake in SHR, indicating that salt status contributes to the maintenance of hypertension in these rats [94]. In this rat model, multiple mechanisms possibly causative of insulin resistance were reported. Significantly reduced glucose uptake in isolated adipocytes [95], reduced GLUT-4 mRNA in cardiac muscle [96], decreased insulin-induced insulin receptor autophosphorylation and IRS-1 phosphorylation in muscle and liver [97], and increased insulin-induced glycogen synthase activity [98] were observed in SHR as compared to WKY. Other studies on SHR have suggested a putative contribution to insulin resistance of adiponectin [99] and folate deficiency [100] or activation of inflammatory markers [101] and retinol-binding protein [102]. Furthermore, indirect evidence of additional mechanisms was obtained with the use of a low-carbohydrate diet [103] and administration of dipeptidyl peptidase-4 inhibitors [104] or angiotensin II receptor antagonists [105].

**Figure 2:**
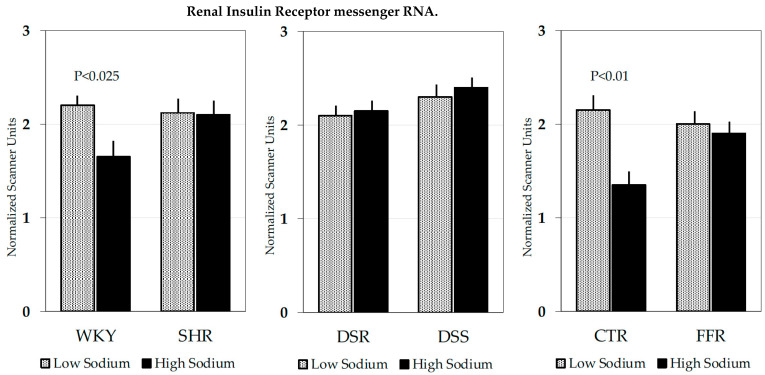
Bar graphs showing mean ± standard error of insulin receptor mRNA in total kidney extracts that were obtained in separated experiments from Wistar-Kyoto (WKY) and Spontaneously Hypertensive (SHR) rats; Dahl salt-resistant (DSR) and Dahl salt-sensitive (DSS) rats; Control (CTR); and Fructose-fed (FFR) rats. Rats were fed low-sodium (shaded bars) or high-sodium (black bars) diets for 2 weeks. Optical density was determined on Northern blots and is expressed as scanner units normalized for 28S ribosomal RNA. In WKY and Control rats, the high-sodium diet induced a significant decrease in insulin receptor gene expression that was not found in SHR and FFR rats.

We measured the expression of the insulin receptor gene by measuring mRNA levels in the liver and skeletal muscle of SHR and WKY rats [106]. Hepatic insulin receptor mRNA levels were significantly decreased in SHR, but no difference was found in skeletal muscle. To further explore the insulin-sodium interaction in SHR, we examined the effects of different dietary sodium contents on the metabolic and renal response to hyperinsulinemic-euglycemic clamp in SHR and WKY [107]. On a low-salt diet, the glucose infusion rate required to maintain normal blood glucose during the clamp was significantly lower in SHR than WKY, and urinary sodium excretion was comparably reduced in both groups. On a high-salt diet, the glucose infusion rate during the hyperinsulinemic clamp decreased significantly in WKY but remained unchanged in SHR. In SHR and WKY rats that were fed the low-sodium and high-sodium diets, we also measured renal insulin receptor density and gene expression. On the low-salt diet, both insulin receptor density and mRNA were comparable in the kidney of SHR and WKY, but on the high-salt diet, only WKY rats exhibited the expected reduction of insulin receptor density and mRNA levels (Table 1 and Figure 2). Although changes in insulin receptor mRNA levels do not necessarily demonstrate changes in gene transcription, these findings strongly suggest the existence of a defective response of the insulin receptor gene to manipulations of dietary sodium in SHR. These observations indicate that SHR have lost the loop mechanism that blunts the antinatriuretic effect of insulin when the extracellular fluid volume is expanded by high sodium feeding. This defect could bolster sodium retention resulting in the development/maintenance of high blood pressure in SHR.

#### 4.3.2. Dahl Hypertensive Rats

Similar to SHR, Dahl hypertensive rats have a form of genetically induced hypertension that is completely dependent on dietary salt intake and is associated with insulin resistance and increased insulin response to an oral glucose load [108,109]. Early studies demonstrated that chronic insulin infusion in Dahl salt-sensitive (DSS) rats caused sodium retention and increased blood pressure; responses that were not seen in Dahl salt-resistant (DSR) control animals [110]. Conversely, assessment of the effects of sodium loading/restriction on insulin sensitivity in this rat strain provided inconsistent results [111,112]. Insulin receptor binding and mRNA levels were measured in the tissues of Dahl hypertensive rats under different sodium-containing diets. Receptor numbers and tissue distribution were comparable in DSS and DSR rats and insulin binding was not modified by the dietary sodium content (Table 1 and Figure 2). Insulin receptor mRNA levels in the liver, skeletal muscle, and kidney were also comparable in DSS and DSR that were fed either a low-salt or high-salt diet [113]. These results indicate that resistance to insulin effects in this rat strain does not occur at the receptor level. Later studies conducted with the euglycemic-hyperinsulinemic clamp demonstrated that salt loading for four weeks induced hypertension and reduced glucose uptake by the isolated soleus muscle in DSS but not DSR rats [114]. In this study, however, despite evidence of reduced sensitivity to insulin, insulin receptor autophosphorylation, phosphorylation of IRS, activation of PI3K, and phosphorylation of AKT were comparable in DSS and DSR rats. This led us to hypothesize that in Dahl hypertensive rats the tissue response to insulin might be impaired in steps located further downstream in the insulin signaling pathway.

#### 4.3.3. Fructose-Fed Rats

Chronic fructose feeding in rats causes hypertension associated with insulin resistance and hyperinsulinemia [115]. Many studies reported increased plasma glucose and insulin levels after a glucose load in fructose-fed rats as compared to controls, and experiments conducted with the use of the hyperinsulinemic-euglycemic clamp showed that fructose-fed rats had significantly reduced glucose disposal rate [116]. In these rats, normalization of circulating insulin levels by exercise training, administration of insulin sensitizers (metformin, troglitazone), or somatostatin were shown to reduce blood pressure, strongly suggesting a causative role of insulin [116]. Early studies suggested that in fructose-fed rats insulin resistance is caused by the reduced ability of insulin to inhibit neoglucogenesis in the liver, while glucose uptake by the skeletal muscle was apparently unchanged [117].

Additional studies were conducted to identify which steps of insulin action are impaired in fructose-fed rats. To investigate whether insulin receptors contribute to changes in glucose metabolism and how receptors are regulated in different tissues, insulin receptor binding and gene expression were measured in the tissues of rats that were fed either standard rat chow or chow containing 66% fructose [118]. Insulin receptor number and mRNA levels were significantly lower in the muscle and liver of fructose-fed rats than in controls, but no difference was found in the kidney. Additionally, abnormalities of insulin action at the post-receptor level were investigated and initial observations did not report significant changes in fructose-fed rats [119]. Bezerra et al. [120] investigated the levels and phosphorylation of the insulin receptor and IRS-1, and the activity of PI3K and phosphotyrosine phosphatase in the liver and muscle of rats fed standard or fructose-enriched chow. No differences were observed in insulin receptor and IRS-1 protein levels, but insulin receptor autophosphorylation, insulin-stimulated IRS-1 phosphorylation, and IRS-1 association with PI3K were all significantly reduced in both muscle and liver of fructose-fed rats.

Studies of sodium dependence in carbohydrate-induced hypertension suggested that blood pressure does not raise when rats are maintained on a low-salt diet [121]. We maintained fructose-fed rats and control animals on a low-sodium and high-sodium chow and measured the rate of glucose infusion during the hyperinsulinemic-euglycemic clamp [122]. When rats were fed the low-salt diet, the glucose disposal rate during the clamp was lower in fructose-fed than in control rats. The high-salt diet significantly decreased the glucose disposal rate during the hyperinsulinemic clamp in control rats but not in fructose-fed rats. On the low-salt diet, insulin receptor density and mRNA levels were lower in the skeletal muscle of fructose-fed than control rats, whereas the high-salt diet significantly decreased insulin receptor density and mRNA levels only in the skeletal muscle of control rats. These findings indicate that reduced glucose disposal rate during the hyperinsulinemic clamp is caused by lower insulin receptor expression in the skeletal muscle of fructose-fed rats fed a low-salt diet, but no change in insulin sensitivity occurs in these rats when fed a high-salt diet.

The renal response of fructose-fed rats to hyperinsulinemia was also examined and as expected, on the low-salt diet a significant decrease in urinary sodium excretion was observed during the clamp that was comparable to that found in control rats [122]. Consistently, on the low-salt diet, renal insulin receptor number and mRNA levels were comparable in fructose-fed and control rats. When rats were maintained on the high-salt diet, the antinatriuretic response to the hyperinsulinemic clamp was reduced in control rats but did not change in the fructose-fed animals. Renal insulin receptor analysis in rats maintained on the high-salt diet revealed a significant decrease in number and mRNA levels in control but not fructose-fed animals (Table 1 and Figure 2), and of particular interest, an inverse relationship was observed between dietary sodium content and renal insulin receptor mRNA levels in control but not fructose-fed rats. Thus, similar to SHR, sodium dependence of hypertension in fructose-fed rats could be related to the loss of the loop mechanism that limits insulin-induced antinatriuresis when extracellular fluid volume is expanded by high-sodium feeding.

## 5. Conclusions

**Figure 3:**
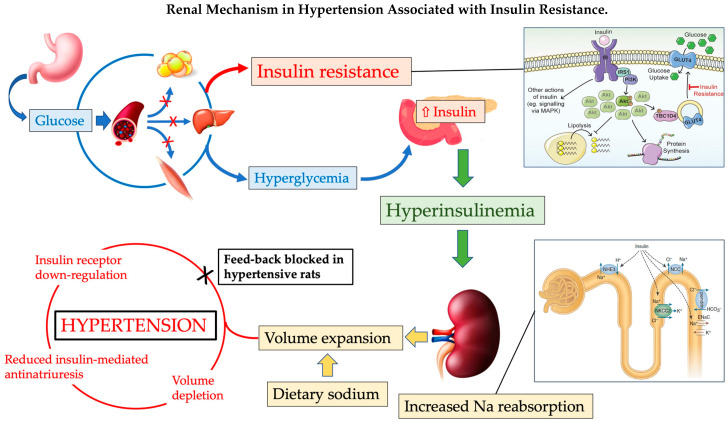
Reduced receptor responsiveness in tissues primarily involved in the metabolic effects of insulin causes decreased glucose uptake and, in turn, leads to a compensatory response of the pancreatic islets with hyperinsulinemia. Excess circulating insulin acting on renal receptors increases sodium reabsorption with extracellular volume expansion. In normal conditions, volume expansion down-regulates renal insulin receptors restoring a normal volume status. In some rat strains of hypertension associated with insulin resistance, the feedback mechanism that reduces insulin-mediated antinatriuresis is defective thereby leading to the persistence of volume expansion and increase in blood pressure.

In this narrative review, we have summarized the current understanding of the potential contribution of insulin-related mechanisms to blood pressure increase, with a specific focus on renal mechanisms and on the role of sodium sensitivity. Using rat models in which hypertension is associated with insulin resistance and like other conditions associated with insulin resistance, abnormalities in insulin-related pathways have been identified at different steps. In some of these rat models, modulation of insulin sensitivity in the kidney is unresponsive to manipulations of dietary sodium, suggesting a defect in the transcriptional response of the insulin receptor gene to volume expansion that could contribute to blood pressure increase (Figure 3).

Awareness of the importance of insulin resistance and hyperinsulinemia in hypertension warrants further efforts to obtain a better understanding of the cellular mechanisms underlying this condition. Knowledge of these mechanisms would help us to develop more appropriate treatments for those patients in whom hypertension is associated with insulin resistance.

## Figures and Tables

**Table 1:** Insulin binding characteristics in kidneys of rat strains with hypertension associated with insulin resistance and hyperinsulinemia maintained on a low-sodium or high-sodium diet.

WKY		SHR	
Low-sodium	High-sodium	Low-sodium	High-sodium
6.9 ± 1.0	4.5 ± 0.7 *	6.5 ± 1.2	6.4 ± 1.1
DSR		DSS	
Low-sodium	High-sodium	Low-sodium	High-sodium
7.5 ± 1.0	5.9 ± 1.6	6.3 ± 1.3	6.8 ± 1.6
CTR		FFR	
Low-sodium	High-sodium	Low-sodium	High-sodium
6.5 ± 1.2	3.8 ± 1.7 *	6.4 ± 1.1	6.5 ± 1.7

Values are expressed as the maximum binding capacity (Bmax; mean ± standard deviation) of the high-affinity insulin receptor site that was calculated by Scatchard analysis on whole renal sections. WKY: Wistar-Kyoto rats, SHR: Spontaneously Hypertensive rats; DSR: Dahl salt-resistant rats; DSS: Dahl salt-sensitive rats; CTR: controls to fructose-fed rats; FFR: Fructose-Fed rats. * *p* < 0.05.

## Data Availability

Not applicable.
